# Adaptive platform trials: testing many interventions under one roof

**DOI:** 10.36416/1806-3756/e20260500

**Published:** 2026-11-09

**Authors:** Bruno L Ferreyro, Cecilia Maria Patino, Juliana Carvalho Ferreira

**Affiliations:** 1. Methods in Epidemiologic, Clinical, and Operations Research-MECOR-program, American Thoracic Society/Asociación Latinoamericana del Tórax, Montevideo, Uruguay.; 2. Interdepartmental Division of Critical Care Medicine, University of Toronto, Toronto, ON, Canada; 3. Department of Medicine, Sinai Health System, Toronto, ON, Canada; 4. Department of Preventive Medicine, Keck School of Medicine, University of Southern California, Los Angeles (CA) USA.; 5. Divisão de Pneumologia, Instituto do Coração, Hospital das Clínicas Faculdade de Medicina, Universidade de São Paulo, São Paulo (SP), Brasil.

## PRACTICAL SCENARIO

An intensivist, two years out of her fellowship, cares for adults with acute hypoxemic respiratory failure in the ICU; she is interested in various key research questions that directly apply to her patients: 1) which noninvasive respiratory support works best (i.e., high flow nasal oxygen or noninvasive ventilation by either a helmet or face mask interface); 2) whether awake prone positioning improves outcomes; and 3) whether her patients should receive aggressive oral nutrition. Testing each question in a separate two arm trial would take several years, duplicate infrastructure, and ‘compete’ for the same patient population. Her mentor suggests a single platform trial, run under one master protocol, in which each patient can be randomized to several interventions at once and new ones can be added over time.

What is an adaptive platform trial? 

A platform trial is a randomized trial built on a master protocol, under which multiple interventions for one disease or syndrome are evaluated simultaneously and/or over time. Interventions can be added or removed from the platform trial according to prespecified decision rules.^1^ Not every platform trial is adaptive; some run under a master protocol with a fixed design, meaning that the treatment arms, allocation ratios, and sample size are set in advance and are not changed in response to accumulating trial data.^1^ Adaptive platform trials differ from traditional trials in two ways: they use a master rather than a standalone protocol and have adaptive rather than fixed design features.^1^ Adaptations follow prespecified rules informed by accumulating data: interim analyses, often Bayesian, stop arms for benefit or futility, and allocation can shift toward promising arms (Figure 1).

**Figure 1:**
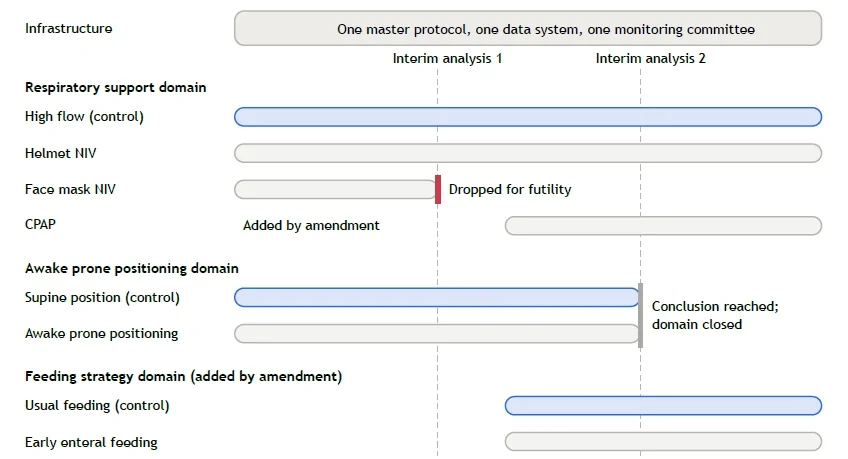
Hypothetical adaptive platform trial for acute hypoxemic respiratory failure. Structure of a hypothetical adaptive platform trial for acute hypoxemic respiratory failure. Domains share one infrastructure, and a patient can be randomized in more than one domain at once. Within each domain, arms share a control group; prespecified interim analyses drop arms for futility or close a domain once a conclusion is reached, and arms or domains added by amendment are compared only with concurrent controls. Results shown are illustrative.

Think of a soccer tournament in which many teams play to find the best one. Nobody would build a new stadium for every match; all the games are played in the same stadium, weaker teams leave the tournament, and new teams can join. In the same way, an adaptive platform trial uses one infrastructure for recruitment, allocation, outcome assessment, and analysis across many trials, run one after another or at the same time.^2^ Active arms often share a common control group, which reduces the total sample size, and a new intervention can join through a protocol amendment instead of a new trial. Respiratory medicine has led the way: during the COVID-19 pandemic, platform trials enrolling hospitalized patients with COVID-19 tested several treatments in parallel, and one demonstrated the benefit of dexamethasone within a few months of the outbreak.^2^

Combining a master protocol with adaptive features also has pitfalls. Adaptive designs are complex, require extensive simulations before enrollment, and remain unfamiliar to many clinicians and funders.^1^ Patients enrolled before an arm opened may differ from those enrolled later (temporal drift), so comparisons should rely on concurrent controls. Informed consent is harder when patients can be randomized in several domains, and funding a platform, rather than a single trial, is more complex.^1^

Artificial intelligence can help at every stage of an adaptive platform trial. Trials can be embedded electronically in routine care,^2^ computer programs that read clinical notes can find eligible patients in electronic records, and chatbots can help write the code used to test the decision rules. A decision support tool based on artificial intelligence can even be tested as one of the arms. However, these tools make mistakes, such as ‘inventing’ references or misreading records, so researchers must check every output, and a statistician should review every decision rule.

Back to our scenario, the researcher and her colleagues at other centers launch an adaptive platform with two domains, that is, sets of options answering one question: respiratory support with noninvasive ventilation or high flow nasal oxygen, and awake prone positioning. Each patient can be randomized in both (Figure 1).^3^ Nutritional strategies open later as a third domain by amendment, and any support strategy added later is compared only with concurrent controls. As early career investigators, they answer their questions within a shared, well mentored infrastructure instead of building it from scratch.

## KEY MESSAGES

- Adaptive platform trials test several interventions for one disease or syndrome under a single master protocol, sharing a control group and adding or dropping arms and adjusting allocation according to prespecified rules informed by accumulating data.
- Their efficiency comes with complexity: nonconcurrent controls, multiple comparisons, and the need for extensive simulation and close statistical support.
- Informed consent is more complex when patients can be randomized in several domains, and funding a platform that runs for years is harder to secure than funding a single trial.
